# Genomic and transcriptomic survey of bryozoan Hox and ParaHox genes with emphasis on phylactolaemate bryozoans

**DOI:** 10.1186/s12864-023-09826-z

**Published:** 2023-11-24

**Authors:** Ahmed J. Saadi, André Luiz de Oliveira, Kevin M. Kocot, Thomas Schwaha

**Affiliations:** 1 Department of Evolutionary Biology, Unit for Integrative Zoology, University of Vienna, Schlachthausgasse 43, Vienna, A-1030 Austria; 2 Department of Symbiosis, Max-Planck-Institute for Marine Microbiology, Celsiustraße,1, D-28359 Bremen, Germany; 3 Department of Biological Sciences and Alabama Museum of Natural History, University of Alabama, Tuscaloosa, Alabama, 35487 USA

**Keywords:** Bryozoans, Phylactolaemate, Hox and ParaHox genes, Comparative genomic and transcriptomic, Gene duplication

## Abstract

**Background:**

Bryozoans are mostly sessile aquatic colonial invertebrates belonging to the clade Lophotrochozoa, which unites many protostome bilaterian phyla such as molluscs, annelids and brachiopods. While Hox and ParaHox genes have been extensively studied in various lophotrochozoan lineages, investigations on Hox and ParaHox gene complements in bryozoans are scarce.

**Results:**

Herein, we present the most comprehensive survey of Hox and ParaHox gene complements in bryozoans using four genomes and 35 transcriptomes representing all bryozoan clades: Cheilostomata, Ctenostomata, Cyclostomata and Phylactolaemata. Using similarity searches, phylogenetic analyses and detailed manual curation, we have identified five Hox genes in bryozoans (*pb*, *Dfd, Lox5, Lox4* and *Post2*) and one ParaHox gene (*Cdx*). Interestingly, we observed lineage-specific duplication of certain Hox and ParaHox genes (*Dfd, Lox5* and *Cdx*) in some bryozoan lineages.

**Conclusions:**

The bryozoan Hox cluster does not retain the ancestral lophotrochozoan condition but appears relatively simple (includes only five genes) and broken into two genomic regions, characterized by the loss and duplication of serval genes. Importantly, bryozoans share the lack of two Hox genes (*Post1* and *Scr*) with their proposed sister-taxon, Phoronida, which suggests that those genes were missing in the most common ancestor of bryozoans and phoronids.

**Supplementary Information:**

The online version contains supplementary material available at 10.1186/s12864-023-09826-z.

## Background

The Hox and ParaHox gene complements are a set of genes that encode transcription factors with a highly conserved sequence region (60 amino acid homeodomain) [1]. They belong to a class of homeobox genes (Antennapedia-class) which are responsible for the regulation of early embryonic development and are involved in patterning the anterior-posterior body axis in Bilateria [2, 3]. Hox genes were originally discovered in the fruit fly *Drosophila melanogaster* [4, 5] and later reported in vertebrates [6, 7], while the ParaHox cluster (the proposed evolutionary sister of the Hox cluster) was initially described in the invertebrate chordate amphioxus *Branchiostoma floridae* [8]. Genomic and transcriptomic data suggest that Hox and ParaHox genes have arisen by duplication and divergence of an ancestral ProtoHox cluster early in metazoan evolution [9]. Based on sequence similarity, the Hox gene complement can be classified into four major classes: the Anterior class, *Hox3* class, Central class and Posterior class [10]. The genetic architecture of these classes varies across taxa due to duplication, inversion, or gene loss events that took place during evolution [9, 10]. Variation in the expression patterns, number, and sequence of Hox and ParaHox genes correlate with body plan evolution during the radiation of several clades such as Arthropoda, Annelida, Chordata and Mollusca [9, 11–13]. Therefore, reconstructing the evolutionary history and determining the expression patterns of these genes are crucial for better understanding animal evolution and the relationships between genetics and different levels of morphological complexity [14].

One of the major clades of protostome animals is Lophotrochozoa, which is mainly characterized by the trochophore, a free-swimming ciliated larva (Trochozoa) and/or the lophophore feeding organ composed of ciliated tentacles surrounding the mouth (Lophophorata) [15]. The ancestral Hox gene complement of Lophotrochozoa is hypothesized to have been composed of 11 genes (*Hox1*/*lab*, *Hox2*/*pb*, *Hox3, Hox4/Dfd, Hox5/Scr, Lox5, Antp, Lox4, Lox2, Post1 and Post2*) whilst the ParaHox cluster generally comprises three genes (*Gsx*, *Xlox* and *Cdx*) [16]. The Hox and ParaHox gene complements of lophotrochozoans have been examined in a variety of taxa based on genomic or transcriptomic data (for details see [16]). In the lophophorate clades Brachiopoda and Phoronida, it seems that the Hox and ParaHox gene complements retain the ancestral lophotrochozoan condition [17–19]. For example, the Hox cluster in the phoronids (*Phoronis australis* and *P. harmeri*) consists of eight Hox genes, with *Antp*, *Post1* and *Scr* being absent [17, 19]. Likewise, the Hox cluster in brachiopods (*Lingula anatina* and *Terebratalia transversa*) comprises almost a complete set of genes, with only *Post1* missing *in L. anatine and Scr missing in T. transversa* [17, 20]. Additionally, Both Brachiopoda and Phoronida possess a complete set of ParaHox genes [17, 19, 20].

Within Lophotrochozoa, Bryozoa represents a rather large aquatic phylum with more than 6,000 extant species of almost entirely colonial suspension feeders [21, 22]. The evolution of bryozoan diversity might be linked to the role of developmental genes, particularly Hox genes [23]. It has been shown that Hox genes can be recruited and coopted into lineage-specific morphological structures, in addition, to their ancestral role in head-to-tail body plan specification [16, 24]). Although the Hox and ParaHox gene complements of lophotrochozoans have been extensively investigated, in the lophophorate group Bryozoa, the presence or absence of Hox genes has been only examined in a single bryozoan species, *Bugula turrita*, based on a targeted search using polymerase chain reaction (PCR) [25] while the ParaHox genes have never been investigated. The examination of Hox genes in Bryozoa revealed a possible loss of six genes including *Hox1*, *Hox5*, *Antp*, *Lox4*, *Lox2*, and *Post1* [25]. Genomic and transcriptomic studies of bryozoans are limited [26–28] and have not focused on the evolution or the organization of Hox and ParaHox genes.

Our understanding of Hox and ParaHox gene evolution in Bryozoa is currently limited and it is largely based on targeted search of Hox genes using PCR where it is difficult to recover the full Hox cluster. Therefore, a comprehensive investigation of Hox and ParaHox clusters using genomic and transcriptomic data is required to ascertain whether the alleged missing Hox genes are indeed absent from the genomes of bryozoans. In this study, we provide the first examination of Hox and ParaHox genes in bryozoans by analysing four genomes and 35 transcriptomes from representative of all main bryozoan linages: Gymnolaemata, Phylactolaemata and Stenolaemata. Using similarity searches, phylogenetic inferences and a detailed manual curation (i.e., identification of conserved residues and protein motifs), we have identified and confirmed the orthology of five Hox and one ParaHox genes in 35 bryozoan species, providing clustering evidence for Hox genes.

## Results

### **Ho*x*and ParaHox genes in bryozoans**

**Fig. 1:**
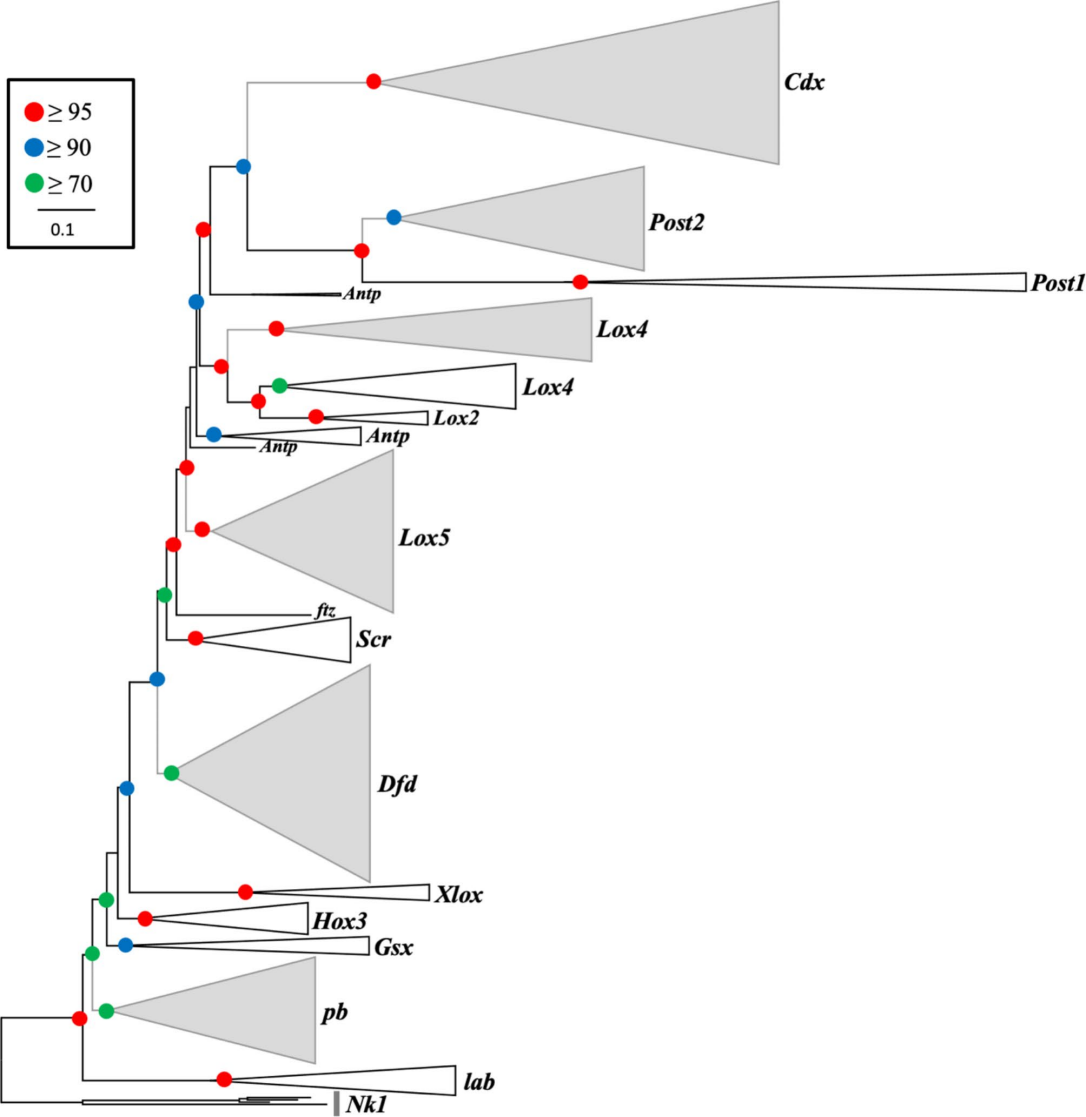
Maximum likelihood phylogeny of Hox and ParaHox genes based on amino acid sequences containing homeodomain and flanking regions of bryozoans and a selection of lophotrochozoan species. ML bootstrap support values are represented by the coloured circles on tree nodes. Clades were collapsed to allow better visibility. Recovered Hox and ParaHox genes in bryozoans are highlighted in grey and their expanded trees are provided in Supplementary Figure S3 -S8. The scale bar indicates amino acid substitutions per site. The homeobox gene Nk1 was used as the outgroup

**Fig. 2:**
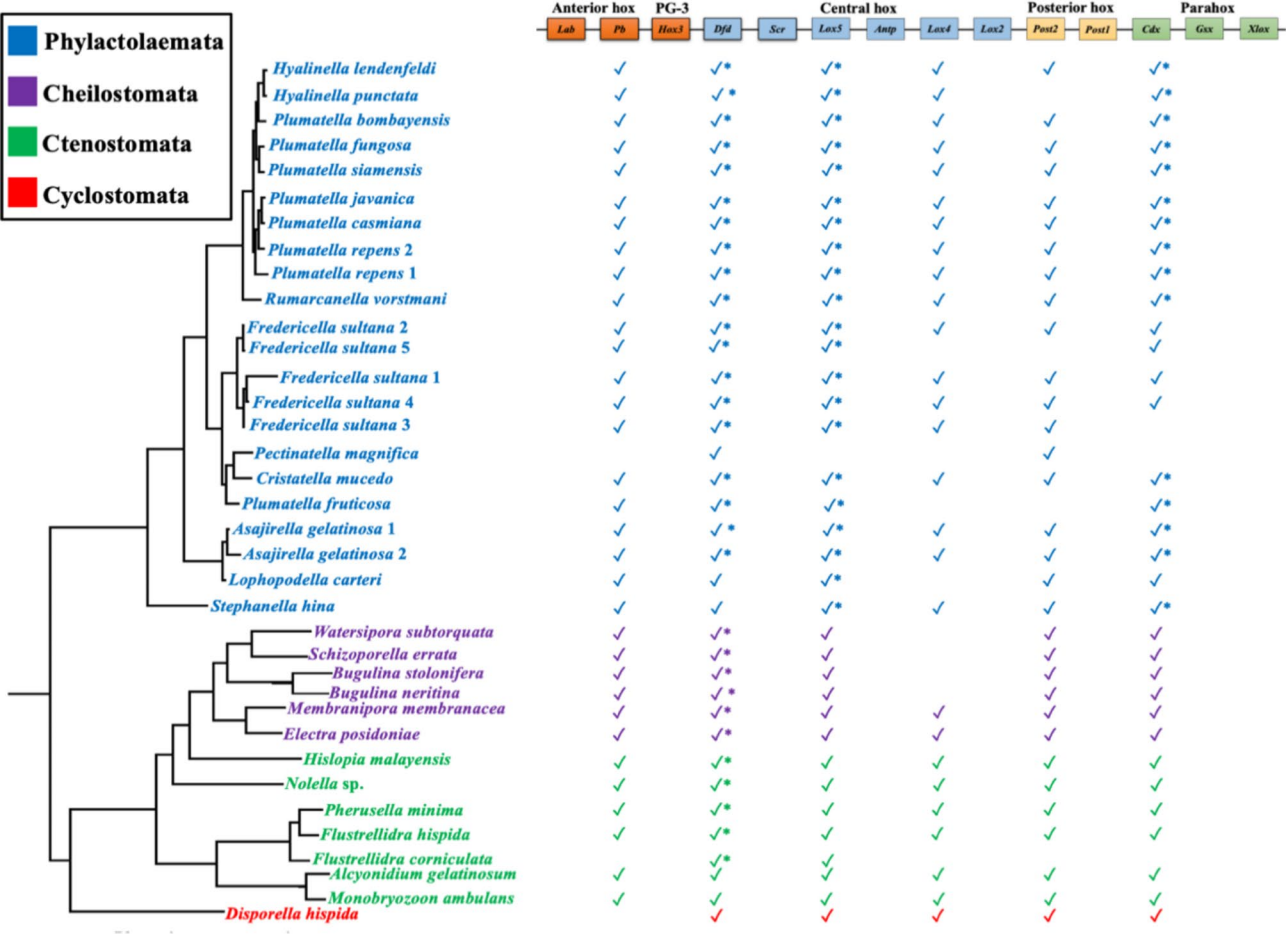
Summary of the Hox and ParaHox genes identified in the 35 bryozoan species studied herein. For comparison, the putative ancestral lophotrochozoan Hox/ParaHox complement is provided. Tree topology follows Saadi et al. [30]. Check signs represent the presences of genes and asterisks indicate duplicated genes

**Fig. 3:**
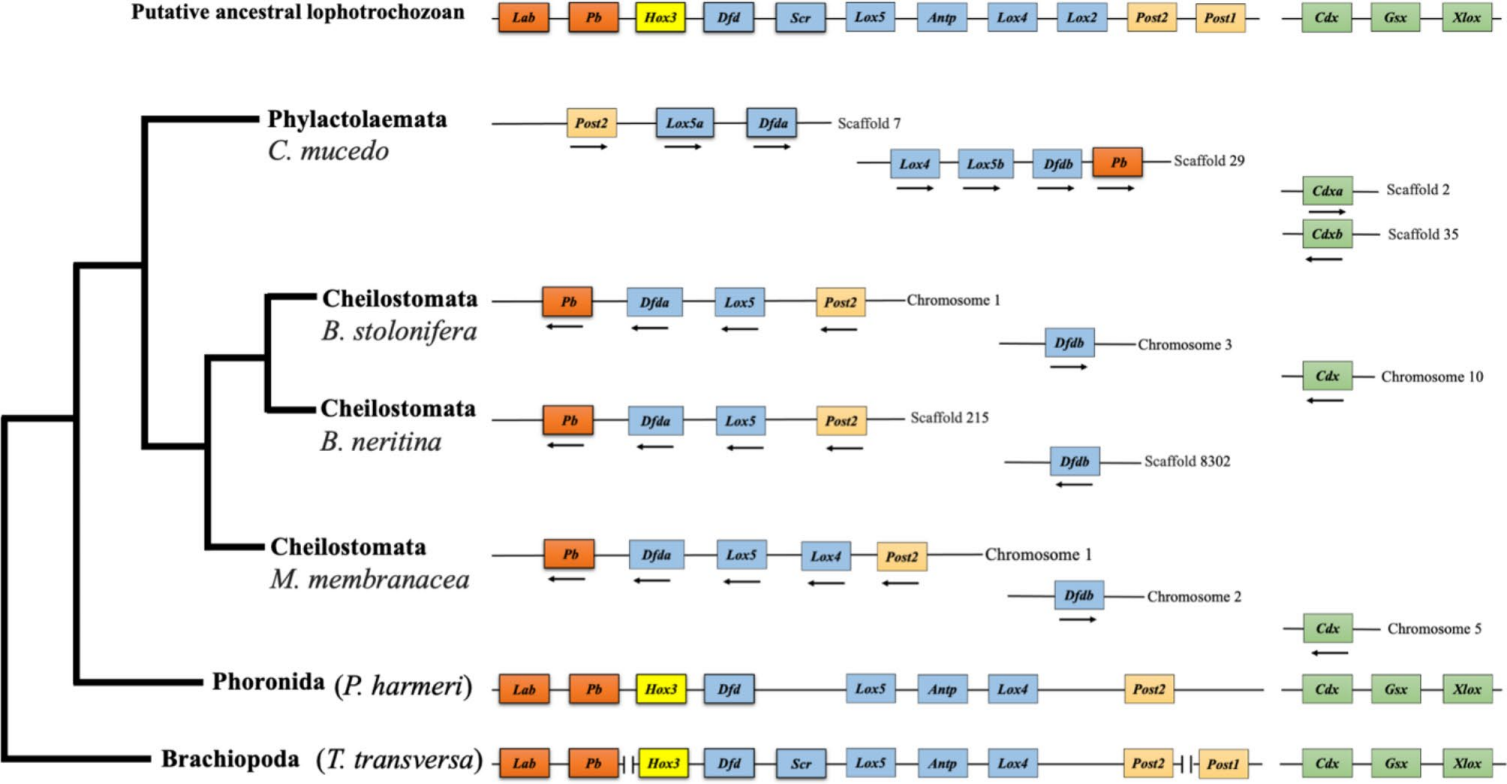
A comparison of Hox and ParaHox clusters among lophophorates. On the left, bryozoan phylogeny with Phoronida and Brachiopoda follows Saadi et al. [30]. On the right, schematic representation of Hox and ParaHox gene complements of Lophophorata (Hox gene clusters of brachiopods and phoronids follows [20]). For comparison, the putative ancestral lophotrochozoan gene toolkit is provided. The coloured boxes indicate the presences of Hox and ParaHox genes. Arrows indicate transcript directions. Hox genes that are hosted within the same chromosome or scaffold are connected by a gray line. The intergenic spaces are not scaled

Assessment of the 35 transcriptomes and the four genomes with BUSCO showed that the majority of the investigated transcriptomes (28) and two genomes have BUSCO values for complete and fragmented sequences above 94%, indicating high completeness scores (Supplementary Figure S1). Of the 11 ancestral Hox genes in Lophotrochozoa, only five candidate orthologues of Hox genes were identified in the bryozoan genomes and transcriptomes. Those genes represent orthologues of the Hox genes *proboscipedia* (*pb*), *Deformed* (*Dfd*), *Lox5*, *Lox4* and *Post2* (Figs. 1, 2 and 3 and Supplementary Figures S2-S8). Of the three genes belonging to the ParaHox complement in Lophotrochozoa, only one candidate orthologue was found (*Cdx*) in our analyses (Figs. 1, 2 and 3, and Supplementary Figures S2 and S8). The recovered Hox and ParaHox genes of bryozoans (except *Lox4*) form distinct clades in our phylogenetic trees with bootstrap support values ≥ 70 (Fig. 1) and posterior probabilities ≥ 0.90 (Supplementary Figure S2). Exact orthology assessment of the *Lox4* gene in bryozoans was not possible based on our phylogenetic analyses (Supplementary Figures S6) as *Lox4* orthologues in bryozoans showed a sister-group relationship to a clade comprising both *Lox2* and *Lox4* genes of non-bryozoan taxa. Therefore, we have relied on the sequence alignment to confirm the identity of *Lox4* in bryozoans and found that the sequence of this gene has most of the diagnostic signature residues of *Lox4* [29] (Supplementary Figure S9) and lacks typical signatures of *Lox2* [29]. For the Hox *Dfd* gene, two gene copies *Dfda* and *Dfdb* were identified in bryozoans, both copies form distinct clades in Phylactolaemata with strong support, whereas *Dfda* is distributed on two separate clades in Myolaemata (Supplementary Figures S4). Likewise, two copies were recovered for the Hox gene *Lox5* (*Lox5a*, *Lox5b*, Supplementary Figures S5) and the ParaHox gene *Cdx* (*Cdxa* and *Cdxb*, Fig. 9) in phylactolaemate bryozoans. These gene copies form well-supported clades in the phylogenetic trees. Our genomic analysis has demonstrated that each copy of the *Dfd*, *Lox5* and *Cdx* genes exists in distinct genomic environments, confirming they are authentic duplicates, and not merely a result of alternative splicing or assembly artifacts.

Finally, orthologues of six Hox genes (*lab*/*Hox1*, *Hox3*, *Scr/Hox5*, *Antp*, *Lox2* and *Post1*) and two ParaHox genes (*Gbx* and *Xlox*) were not identified in the investigated bryozoan genomes or transcriptomes. Additionally, we found that the gene identified by YL Passamaneck and KM Halanych [25] as *Hox3* in *Bugula turrita* is most likely contaminated or misidentified (Supplementary Figure S10).

### Hox cluster organization in bryozoan genomes

We examined the organization of the Hox cluster in four bryozoan species whose genomes are publicly available, three of them belonging to Cheilostomata (*Bugula neritina*, *Bugulina stolonifera* and *Membranipora membranacea*) and one to Phylactolaemata (*Cristatella mucedo*). In the three cheilostome genomes (*B. neritina*, *B. stolonifera and M. membranacea*), Hox genes are located in two different genomic regions (chromosomes or scaffolds), one containing *pb*, *Dfda*, *Lox5* and *Post2* genes (in *M. membranacea* it also includes *Lox4*) and the other one containing the *Dfdb* gene. Hox genes have a consistent order among these three genomes and they share the same transcriptional orientation except *dfdb* in *B. neritina* which is transcribed in a different direction (Fig. 3). In both of *B. stolonifera and M. membranacea* genomes, Hox genes occur on the same chromosome (chromosome two) except *Dfdb* which is located on chromosome three in *B. stolonifera* and on chromosome one in *M. membranacea* (Fig. 3). In the phylactolaemate species *Cristatella mucedo*, Hox genes also fall into two different scaffolds, one harbouring *pb*, *Dfdb*, *Lox5b* and *Lox4* genes and the other containing all the remaining Hox genes (*Dfda*, *Lox5a* and *Post2*), (Fig. 3). The Hox gene order in *C. mucedo* diverge from the other three cheilostome genomes. The first part of the Hox cluster in *C. mucedo* includes three genes in the following order: *Post2*, *Lox5a*, and *Dfda* while the second part contains four genes with the order *Lox4, Lox5b, Dfdb* and *pb* (Fig. 3). Furthermore, in *C. mucedo*, the ParaHox cluster exhibits distinctions compared to the three other cheilostome genomes due to the presence of two copies of the *Cdx* gene, each located on separate genomic regions. It is worth noting that the intron numbers of *Dfdb* and *Lox4* genes in *C. mucedo* also diverge from those in the other cheilostome genomes (Table 1).

**Table 1:** Number of introns (first value) and protein length (second value) of the identified Hox and ParaHox genes in four bryozoan genomes (absent genes = “-”)

Genes Species	*pb*	*dfda*	*dfdb*	*lox5a*	*lox5b*	*lox4*	*post2*	*cdxa*	*cdxb*
***B. neritina***	2/256	2/233	3/231	2/255	-	-	1/265	-	-
***B. stolonifera***	2/264	2/236	3/231	1/259	-	-	1/270	2/254	-
***M. membranacea***	2/251	1/183	3/234	1/242	-	1/77	1/249	2/342	-
***C. mucedo***	2/309	2/258	1/261	1/219	1/259	3/222	1/219	2/213	2/299

## Discussion

In this study, we examined the Hox and ParaHox genes in 35 bryozoan transcriptomes and four genomes representing all bryozoan clades: Cheilostomata, Ctenostomata, Cyclostomata and Phylactolaemata. Five Hox genes (*pb*, *Dfd, Lox5, Lox4* and *Post2*) and one ParaHox gene (*Cdx*) were identified in bryozoans. Our results revealed that bryozoans have experienced significant gene losses in the Hox and ParaHox complements including loss of six Hox genes (*lap*, *Hox3*, S*cr*, *Antp, Lox2* and *Post1*) and two ParaHox (*Gsx* and *Xlox)* genes. Prior to our study, Hox genes had only been investigated in a single bryozoan species [25] in which five Hox genes (*pb*, *Hox3*, *Dfd, Lox5*, and *Post2*) were also identified. However, the *Hox3* gene was not recovered from any bryozoan in our analyses indicating that this gene is evidently missing in bryozoans and that the *Hox3* sequence previously reported by YL Passamaneck and KM Halanych [25] may have been the result of contamination. This is also suggested by our phylogenetic analysis as the previously published *Hox3* gene with non-bryozoan *Hox3* orthologues with strong support.

The *Hox3* gene has been reported in most of the lophotrochozoan linages [16] including the lophophorate groups Brachiopoda [18, 31] and Phoronida [17, 19]. However, specific role of *Hox3* varies between different lophotrochozoan taxa and it has not been fully characterized in most phyla. For example, in the phoronid *Phoronopsis harmeri*, the *Hox3* gene is exclusively expressed in ectodermal cells [17, 19] while in the brachiopod species, *Novocrania anomala* and *Terebratalia transversa*, *Hox3* is expressed in ectodermal and mesodermal cells [31]. Similarly, the *Antp* gene is also missing in bryozoans, but present in many other lophotrochozoan groups [16] including brachiopods and phoronids where it is only ectodermally expressed [17, 19]. Since both *Hox3* and *Antp* genes are missing in bryozoans and present in brachiopods and phoronids, we suggest that those two genes were lost after bryozoans split from their last common ancestor.

Although it has been suggested that *Post1* and *Scr* genes are involved in biomineralization of brachiopods [20, 31], the lack of these two genes in bryozoans is not surprising as the ancestor of bryozoans was soft-bodied [30, 32]. The mineralized skeleton of cheilostomes and stenolaemates has most likely evolved independently in these two clades [33, 34]. Furthermore, the lack of *Post1* and *Scr* genes in bryozoans is shared with phoronids which might indicate that these genes were lost in their last common ancestor. A sister-group relationship between phoronids and bryozoans has been proposed by several phylogenomic studies [35–38] and our finding regarding the shared missing Ho*x* genes in bryozoans and phoronids are supportive of this hypothesis. However, a recent phylogenomic analyses recovered a sister group relationship between Kamptozoa (= Entoprocta) and Bryozoa, which contradicts the monophyly of Lophophorata [39]. In comparison to bryozoans, almost complete sets of Hox genes (10 of 11 Hox genes commonly found in Lophotrochozoans) were identified in kamptozoan transcriptomes and genomes [40]. Thus, the data obtained in our study provides evidence for a closer relationship of bryozoans to phoronids than to kamptozoans.

Interestingly, two of the identified Hox gene orthologues in bryozoans (*Dfd* and *Lox5*) have two copies in most bryozoans. The phylogenetic analyses provide strong support for each copy of those orthologue groups. The duplication of *Dfd* and *Lox5* genes is also confirmed by the genomic analysis since each copy is located on a different genomic region. In the *Dfd* gene, the duplication occurs in three bryozoan linages: Cheilostomata, Ctenostomata and Phylactolaemata while the duplication of *Lox5* gene occurs only in phylactolaemate bryozoans. Two copies of the *Dfd* gene were previously reported in *Bugula turrita* by YL Passamaneck and KM Halanych [25] where they suggested that duplication of *Dfd* gene happened when bryozoans formed an independent lineage. Duplication events in the *Dfd* gene have also been reported in other spiralian lineages including Rotifera (*Adineta vaga*) [13], Annelida (*Perionyx excavatus*) [41] and in the nemertean *Notospermus geniculatus* [17]. Meanwhile, the duplication of *Lox5* gene is less common in lophotrochozoans and has only been shown in the platyhelminths *Dugesia japonica* and *Girardia tigrine* [16].

Overall, since the duplication of the *Dfd* gene occurs in both phylactolaemates and myolaemates, it is probable that this gene has undergone duplication independently after the divergence of bryozoans from their last common ancestor while the duplication of *Lox5* gene seems to be linage specific and occurred in last common ancestor of all phylactolaemates.

ParaHox genes have so far not been characterized in any bryozoan species, and similar to the Hox cluster, the ParaHox complement of bryozoans shows gene duplications and losses. For instance, out of the three ancestral lophotrochozoan ParaHox genes (*Gsx*, *Xlox* and *Cdx*), only *Cdx* gene (Caudal) was recovered in bryozoans. However, *Cdx* has two copies in most of the investigated phylactolaemates with each copy located on a separate scaffold of the *C. mucedo* genome. The duplication of *Cdx* gene in phylactolaemates is probably lineage-specific and could be related to the life cycle of this clade. Many studies have suggested that Caudal in lophotrochozoans is generally expressed in ectodermal and endodermal cells and less likely in mesodermal ones [42–45]. However, the functional characterization of Caudal has been less investigated [46] and recently was studied in one lophotrochozoan species (in the embryo of the mollusc *Tritia*), showing that Caudal is required for development of the hindgut in a mollusc [47]. Functional studies of other ParaHox genes are lacking for the vast majority of lophotrochozoan lineages. In contrast to bryozoans, a complete set of ParaHox genes were identified in the phoronids *Phoronis australis* [17] and *Phoronopsis harmeri* [19]. Losses of ParaHox genes is common in lophotrochozoans (e.g., *Cdx* is missing in the kamptozoan *Loxosomella murmanica*, *Gsx* is absent in the kamptozoans *Loxosomella vivipara* and *Pedicellina cernua* [40], and *Xlox* is missing in the nemertean *Notospermus geniculatus* [17]).

Our genomic information shows that the Hox cluster of the cheilostomes *B. neritina*, *B. stolonifera and M. membranacea* is split into two different genomic loci, with *Dfda* being separated from the major cluster. Similarly, the Hox cluster of the phylactolaemate *Cristatella mucedo* is distributed over two scaffolds, one comprises *pb*, *Dfdb*, *Lox5b* and *Lox4* and the other one includes *Dfda*, *Lox5a* and *Post2*. Dissociation of the Hox cluster is common in lophotrochozoans, for example, the annelids *Capitella teleta* has a split Hox cluster and the leech *Helobdella robusta*, shows a highly fragmented Hox complex [48]. The lack of Hox cluster is also reported in the cephalopod *Octopus bimaculoides* [49], the pacific oyster *Crassostrea gigas* [50], the brachiopod *Terebratalia transversa* [31], and the nemertean *Notospermus geniculatus* [17]. Importantly, the phoronid *Phoronis australis* has one Hox cluster [17]. Although, phoronids preserved the Hox synteny, they also lack some of the Hox genes, including *Lox2*, *Post1* and *Scr*. The secondary loss of Hox genes seems pervasive in bryozoans and phoronids. However, in bryozoans, the synteny appears to be broken as well, suggesting that there are two layers of complexity in bryozoans (Loss and shift of the genomic loci). This might indicate that bryozoan genomes are more dynamic, though further syntenic analyses are necessary to investigate these two lineages.

## Conclusions

Bryozoans do not retrain the ancestral lophotrochozoan Hox and ParaHox clusters, but instead they show rather simple (including only six genes) and broken clusters with significant gene losses but also duplications. Only five Hox genes and one ParaHox gene were identified in bryozoans, which is much fewer than the number identified in their closely relative lophophorate taxa brachiopods and phoronids or even in kamptozoans, which have also been hypothesized to the sister-taxon of bryozoans based on a recent phylogenomic study. In addition, Hox gene duplications have neither been reported in other lophophorates nor in kamptozoans, which might indicate that Hox and ParaHox duplications in certain bryozoan genes are lineage specific. Still, exact details and the extent of these duplications are still unclear as most of our data were obtained from transcriptomes. Bryozoans and phoronids lack *Post1* and *Scr* genes which could be related to soft-body status of their common ancestor as those genes are generally expressed in biomineralizing tissues. Further genomic and transcriptomic studies with more taxon sampling (especially cyclostomes) are needed to determine the presence, expression patterns, and functional significance of Hox and ParaHox genes in bryozoans.

## Materials and methods

### Transcriptome assembly and quality assessment

A total of 35 bryozoan transcriptomes (as either raw sequence reads or assembled transcriptomes) and four genome assemblies were obtained from publicly available data (details of the specimens, GenBank Bioproject accession numbers and sources of specimens are provided in Supplementary Table S1). In order to assemble the raw Illumina reads from transcriptomes, adapters and low-quality reads were first removed from raw sequence reads using Trimmomatic v0.39 [51] with default parameters. The clean reads were *de novo* assembled in Trinity v2.8.4 [52], under default settings. The assembled transcriptomes were then run through Transdecoder.LongOrfs v5.02 to extract all possible coding sequence regions from the transcripts and through Transdecoder.Predict v5.02 with the --single_best_only to select only the single best open reading frames (ORFs) (the longest coding sequence regions within the transcript) (https://github.com/TransDecoder/TransDecoder/; last accessed July 29, 2022). Only ORFs that were at least 80 amino acids long were retained. To reduce redundancy in protein gene sets, CD-HIT v4.8.1 [53] was used with a threshold of 95% global similarity. Finally, the gene content and the completeness of the transcriptomes were assessed with BUSCO v4.1.4 [54] using the pre-defined metazoan Benchmarking set of Universal Single-Copy Orthologs with 954 evolutionary conserved orthologous groups (metazoan_odb10).

### Hox and ParaHox gene sequences identification and orthology assessment

Using Blastp v2.12.0+ [55], bryozoan protein sequences from all transcriptomes were searched against well-curated publicly available metazoan Hox and ParaHox sequences including bryozoan Hox gene candidates. The top three blast hits of each similarity search were analysed and blasted back against GenBank non-redundant protein database to reconfirm the homology. Furthermore, to make sure that the full Hox and ParaHox genes in bryozoans were recovered, Exonerate v2.4.0 [56] with protein2genome model and maximum intron length set to 40 kb was used to scan the whole genome assemblies of four bryozoan species (*Cristatella mucedo*, *Bugula neritina*, *Bugulina stolonifera* and *Membranipora membranacea*), whose genome annotations were not publicly available at the time of these analyses. The longest CDS for each Hox and ParaHox gene was then selected and translated to protein using EMBOSS Transeq online server (https://www.ebi.ac.uk/Tools/st/emboss_transeq/) [57]. The putative bryozoan Hox and ParaHox genes from transcriptomes and genomes were aligned together using MAFFT 7.310 [58] combined with their representative homologs from different metazoan phyla also retrieved from GenBank non-redundant protein database under the following options: --localpair and --maxiterate 1000. All sequences were carefully inspected then manually edited with Aliview v.2022 [59] and trimmed with trimAl (-gt 0.4) [60]. Phylogenetic analyses were performed using Maximum likelihood (ML) and Bayesian inference (BI) methods. The ML analysis was conducted with IQ-TREE2 v2.1.2 [61] using ModelFinder tree search with 1,000 ultrafast bootstraps and SH-aLRT test replicates [62, 63]. For BI analysis, the best substitution model was first selected (JTT + I + G4) using maximum AIC as determined using ModelTest-NG [64]. The BI phylogenetic analysis was performed using MrBayes 3.2.7a [65] with JTT + I + G4 model of amino-acid substitution as determined using maximum AIC as implemented in ModelTest-NG [64]. Two independent runs with four chains of Markov Chain Monte Carlo (MCMC) algorithm were used to explore the tree space. BI analysis was conducted for 10 million generations sampled every 100 generations. The first 25% of samples were discarded as burn-in and the remaining trees were used to calculate posterior probability values and to build the consensus tree. The final ML and BI trees were visualized in Figtree v1.4.4 (http://tree.bio.ed.ac.uk/software/figtree/). In case of gene orthology was not confirmed based on phylogenetic analyses (i.e., gene did not form a monophyletic group), the multiple sequence alignment was searched for the presence of diagnostic residues/motifs in the homeodomain and in the flanking regions based on de Rosa et al. (1999).

Finally, to determine Hox and ParaHox gene locations, transcriptional orientations, intron number and lengths in four bryozoan species whose genomes are publicly available (without annotation), we aligned the identified Hox and ParaHox genes of those four species back to their respective genomes using Exonerate with protein2genome as described above.

### Electronic supplementary material

Below is the link to the electronic supplementary material.

Supplementary Material 1

## Data Availability

The data presented in this study including the alignment of the metazoan Hox and ParaHox sequences, the alignment of the identified Hox and ParaHox genes in bryozoans before and after trimming and the full trees are available from figshare: https://figshare.com/s/0ecf2eee0d06fd32c458.
