# Supplementary material for: Genomic and transcriptomic survey of bryozoan Hox and ParaHox genes with emphasis on phylactolaemate bryozoans
Source: BMC Genomics. 2023 Nov 24;24:711. doi: 10.1186/s12864-023-09826-z (PMC10675955; doi:10.1186/s12864-023-09826-z)
Supplement: Supplementary file 1 — Supplementary Material 1 [file 12864_2023_9826_MOESM1_ESM.docx]

**Supplementary material**

**Genomic and transcriptomic survey of bryozoan Hox and ParaHox genes with emphasis on phylactolaemate bryozoans**

Ahmed J. Saadi^1^, André Luiz de Oliveira^2^, Kevin M. Kocot^3^ and Thomas Schwaha^1^

^1^Department of Evolutionary Biology, Unit for Integrative Zoology, University of Vienna, Schlachthausgasse 43, A-1030 Vienna, Austria.

^2^Department of Symbiosis, Max-Planck-Institute for Marine Microbiology, Celsiustraße,1 D-28359 Bremen, Germany

^3^Department of Biological Sciences and Alabama Museum of Natural History, University of Alabama, Tuscaloosa, Alabama, 35487, USA.

$ corresponding author

ahmed.saadi@univie.ac.at

**Table S1**. Details of the specimens, GenBank Sequence Read Archive (SRA) accession numbers and sources of publicly available sequences.

| **Species** | **Accession number or download source** | **Reference** |
| --- | --- | --- |
| **Phylactolaemata** | | |
| *Asajirella gelatinosa* 1 (Oka, 1891) | NCBI, SRA, SAMN25648383 | (Saadi et al., 2022) |
| *Asajirella gelatinosa* 2 (Oka, 1891) | NCBI, SRA, SAMN25648384 | (Saadi et al., 2022) |
| *Cristatella mucedo* Cuvier, 1789 | NCBI, SRA, SAMN25648378 | (Saadi et al., 2022) |
| *Cristatella mucedo* Cuvier, 1789 | WPIO00000000 | (Kutyumov et al., 2021) |
| *Fredericella sultana* 1 Blumenbach, 1779 | NCBI, SRA, SAMN25648379 | (Saadi et al., 2022) |
| *Fredericella sultana* 2 Blumenbach, 1779 | NCBI, SRA, SAMN25648380 | (Saadi et al., 2022) |
| *Fredericella sultana* 3 Blumenbach, 1779 | NCBI, SRA, SAMN25648381 | (Saadi et al., 2022) |
| *Fredericella sultana* 4 Blumenbach, 1779 | NCBI, SRA, SAMN25648382 | (Saadi et al., 2022) |
| *Fredericella sultana* 5 Blumenbach, 1779 | NCBI, SRA, SRP198874 | (Kumar et al., 2020) |
| *Hyalinella lendenfeldi* (Ridley, 1886) | NCBI, SRA, SAMN25648387 | (Saadi et al., 2022) |
| *Hyalinella punctata* Vorstman, 1928 | NCBI, SRA, SAMN25648388 | (Saadi et al., 2022) |
| *Lophopodella carteri* (Hyatt, 1866) | NCBI, SRA, SAMN25648385 | (Saadi et al., 2022) |
| *Pectinatella magnifica* (Leidy, 1851) | NCBI, SRA, SAMN25648386 | (Saadi et al., 2022) |
| *Plumatella bombayensis* Annandale, 1910 | NCBI, SRA, SAMN25648389 | (Saadi et al., 2022) |
| *Plumatella casmiana* Oka, 1907 | NCBI, SRA, SAMN25648390 | (Saadi et al., 2022) |
| *Plumatella fungosa* (Pallas, 1768) | NCBI, SRA, SAMN25648392 | (Saadi et al., 2022) |
| *Plumatella fruticosa* Allmann, 1844 | NCBI, SRA, SAMN25648393 | (Saadi et al., 2022) |
| *Plumatella javanica* Kraepelin, 1906 | NCBI, SRA, SAMN25648394 | (Saadi et al., 2022) |
| *Plumatella repens* 1 (Linnaeus, 1758) | NCBI, SRA, SAMN25648395 | (Saadi et al., 2022) |
| *Plumatella repens* 2 (Linnaeus, 1758) | NCBI, SRA, SAMN25648395 | (Saadi et al., 2022) |
| *Plumatella siamensis* Wood, 2006 | NCBI, SRA, SAMN25648397 | (Saadi et al., 2022) |
| *Plumatella* sp. | NCBI, SRA, SAMN25648399 | (Saadi et al., 2022) |
| *Rumarcanella vorstmani* (Toriumi, 1952) | NCBI, SRA, SAMN25648398 | (Saadi et al., 2022) |
| *Stephanella* cf. *hina* Oka, 1908 | NCBI, SRA, SAMN25648400 | (Saadi et al., 2022) |
| **Stenolaemata: Cyclostomata** | | |
| *Disporella hispida* (Fleming, 1828) | NCBI, SRA, SAMN25648401 | (Saadi et al., 2022) |
| **Gymnolaemata: Cheilostomata** | | |
| *Bugula neritina* | ASM1079987v2 | (Rayko et al., 2020) |
| *Bugulina stolonifera* (Ryland, 1960) | https://doi.org/10.7910/DVN/SDJZ4X | (Treibergs and Giribet, 2020) |
| *Bugulina stolonifera* (Ryland, 1960) | CAKXYU010000000 | (Wood et al., 2023) |
| *Electra posidoniae* Gautier, 1954 | SAMN25648403 | (Saadi et al., 2022) |
| *Membranipora membranacea* (Linnaeus, 1767) | NCBI, SRA, SRR2131259 | (Laumer et al., 2015) |
| *Membranipora membranacea* (Linnaeus, 1767) | CAJZBW000000000 | (Bishop et al., 2023) |
| *Schizoporella errata* (Waters, 1878) | NCBI, SRA, SRR11784297 | (Santagata, 2021) |
| *Watersipora subtorquata* (d'Orbigny, 1852) | NCBI, SRA, SRR11783869 | (Santagata, 2021) |
| **Gymnolaemata: Ctenostomata** | |  |
| *Alcyonidium gelatinosum* (Linnaeus, 1761) | NCBI, SRA, SAMN25648407 | (Saadi et al., 2022) |
| Flustrellidra corniculata (Smitt, 1872) | NCBI, SRA, SRR9667736 | Laumer *et al*. (Laumer et al., 2019) |
| *Flustrellidra hispida* (Fabricius, 1780) | NCBI, SRA, SAMN25648408 | (Saadi et al., 2022) |
| *Hislopia malayensis* Annandale, 1916 | NCBI, SRA, SAMN25648404 | Saadi et al. (Saadi et al., 2022) |
| *Monobryozoon ambulans* Remane, 1936 | NCBI, SRA, SRR25585376 | un published |
| *Nolella* sp*.* | NCBI, SRA, SAMN25648405 | (Saadi et al., 2022) |
| *Paludicella articulata* (Ehrenberg, 1831) | NCBI, SRA, SRR25585375 | un published |
| *Pherusella minima* Decker, Gordon, Spencer Jones & Schwaha, 2021 | NCBI, SRA, SAMN25648406 | Saadi et al. (Saadi et al., 2022) |

**
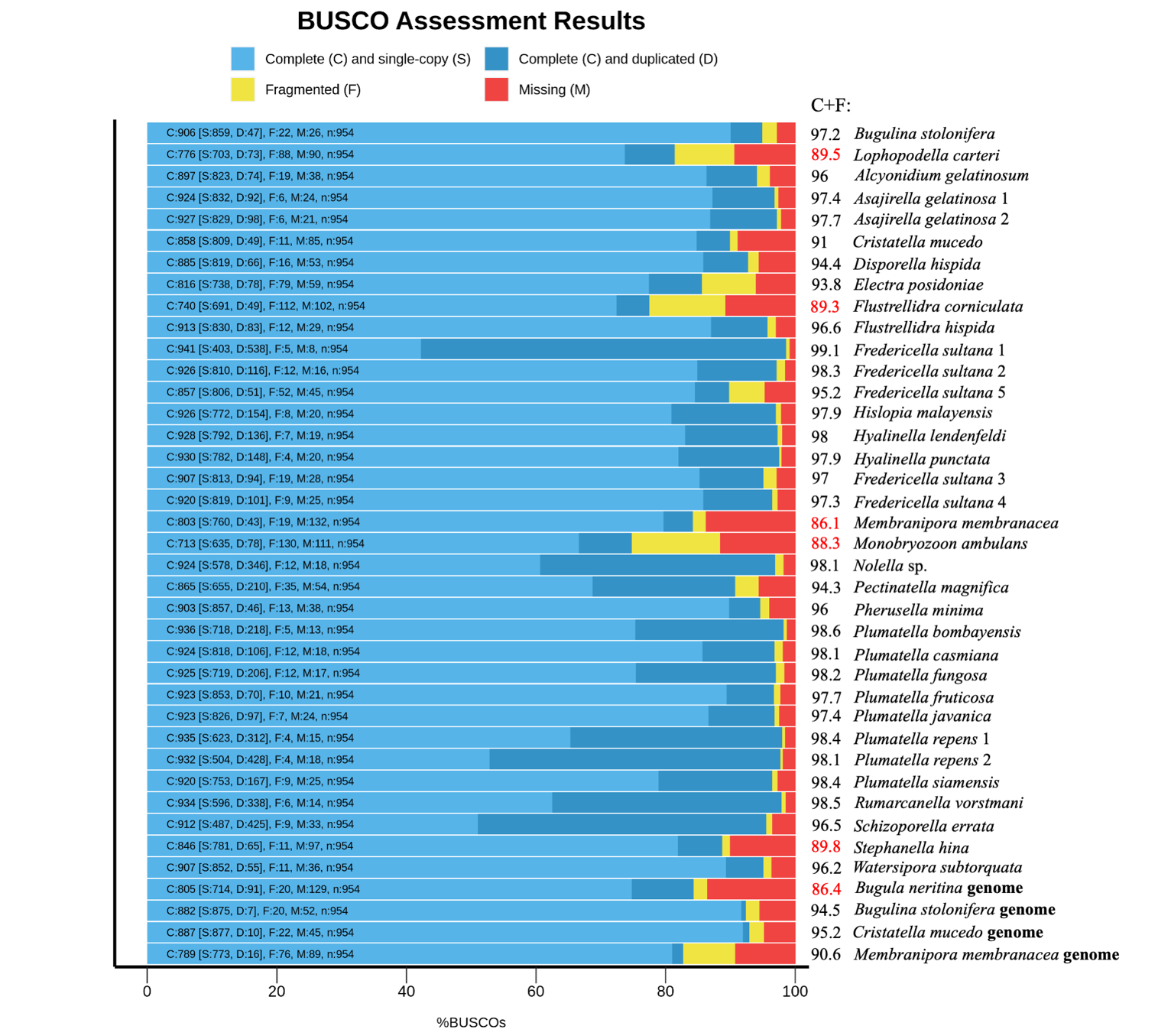
**

Figure S1. BUSCO completeness of the 35 transcriptomes and four genomes based on pre-defined metazoan Benchmarking set of Universal Single-Copy Orthologs with 954 evolutionary conserved orthologous groups (metazoan_odb10). Colours in the bars represent the different categories of the identified BUSCO genes.


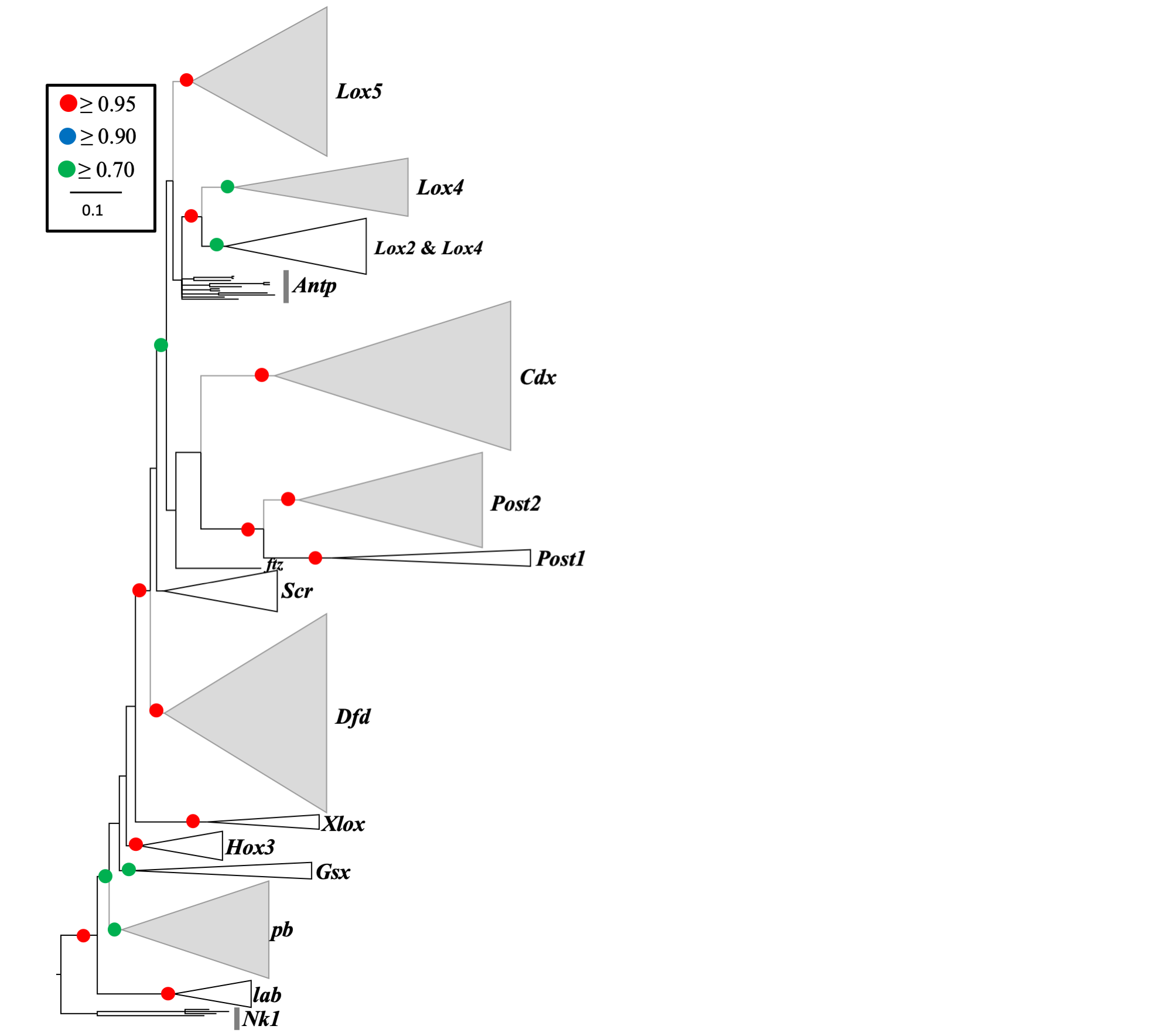


**Figure S2:** Bayesian phylogeny of Hox and ParaHox genes from amino acid sequences containing homeodomain and flanking regions of bryozoans and selected lophotrochozoan species. Bayesian posterior probabilities are represented by the coloured circles on tree nodes. Clades were collapsed to allow better visibility. Recovered Hox and ParaH*ox* genes in bryozoans are highlighted in grey. The scale bar indicates amino acid substitutions per site. The homeobox genes Nk1 were used as outgroup.


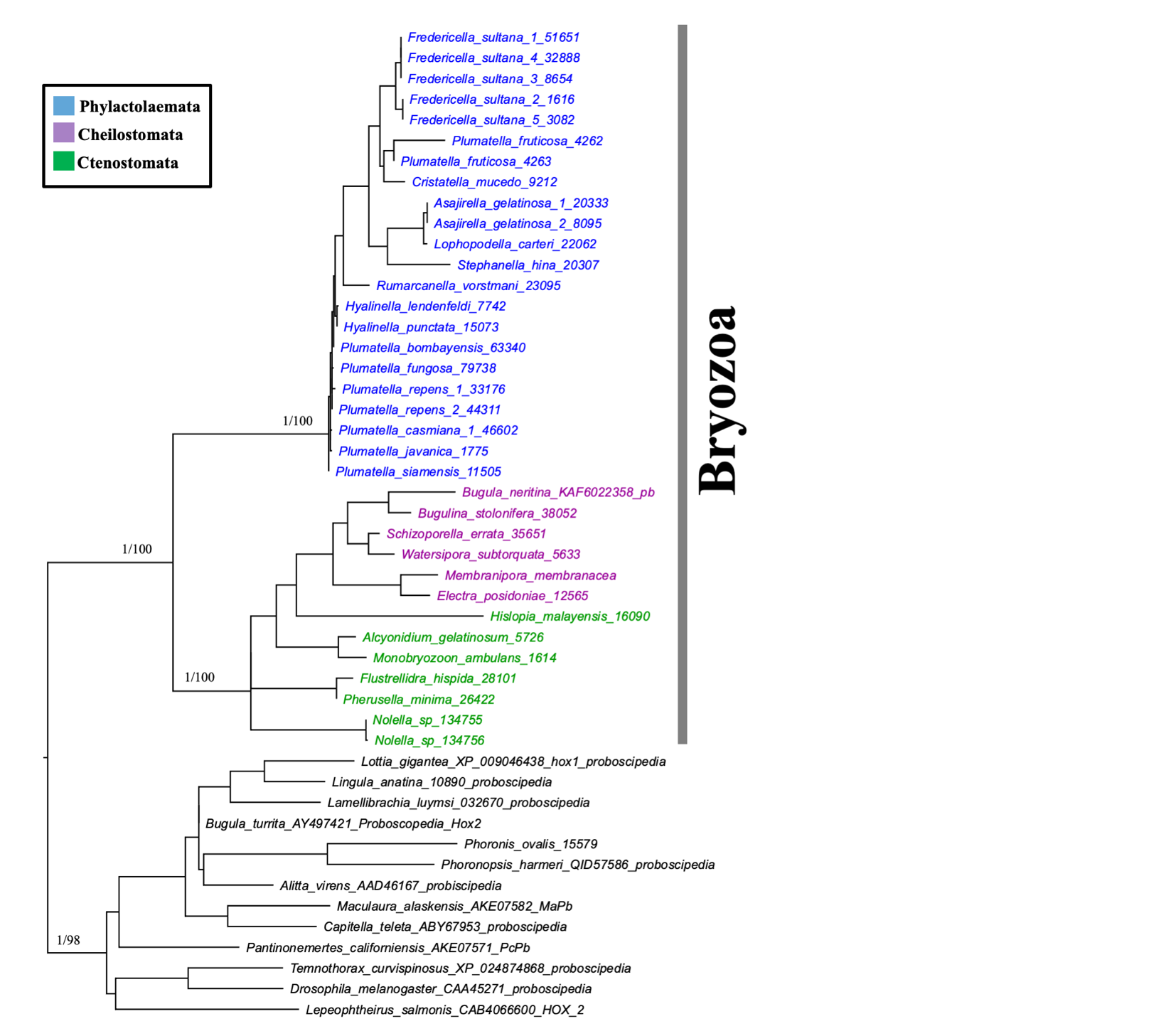


**Figure S3:** Expanded tree inference of *pb* gene (derived from Fig. 1). Bayesian posterior probabilities and bootstrap support values are shown for important clades, respectively. Accession numbers for NCBI database are displayed after the species names.


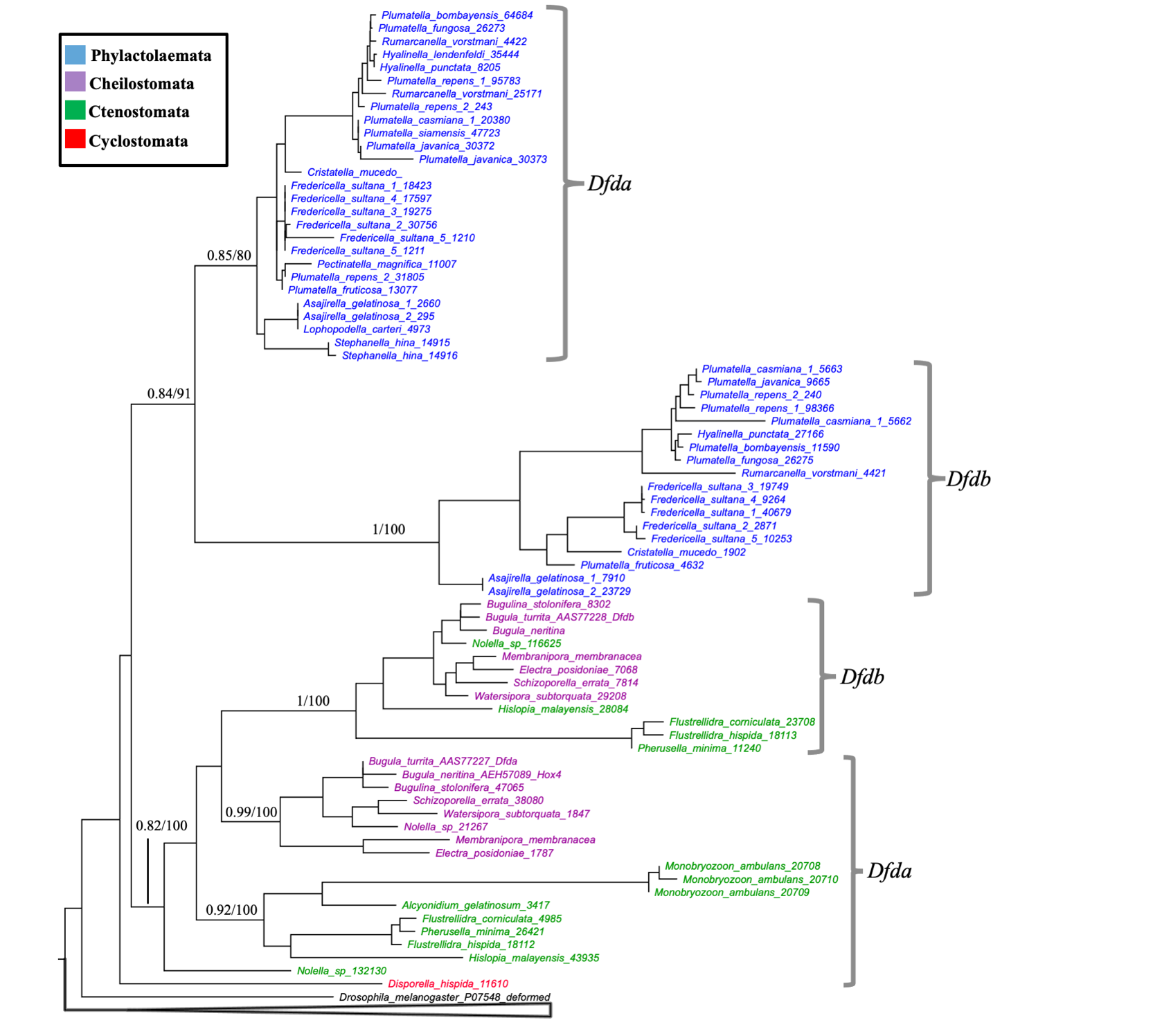


**Figure S4:** Expanded tree inference of *Dfd* gene (derived from Fig. 1). Bayesian posterior probabilities and bootstrap support values are shown for important clades, respectively. Accession numbers for NCBI database are displayed after the species names. Non bryozoan taxa were collapsed for easier visualization.


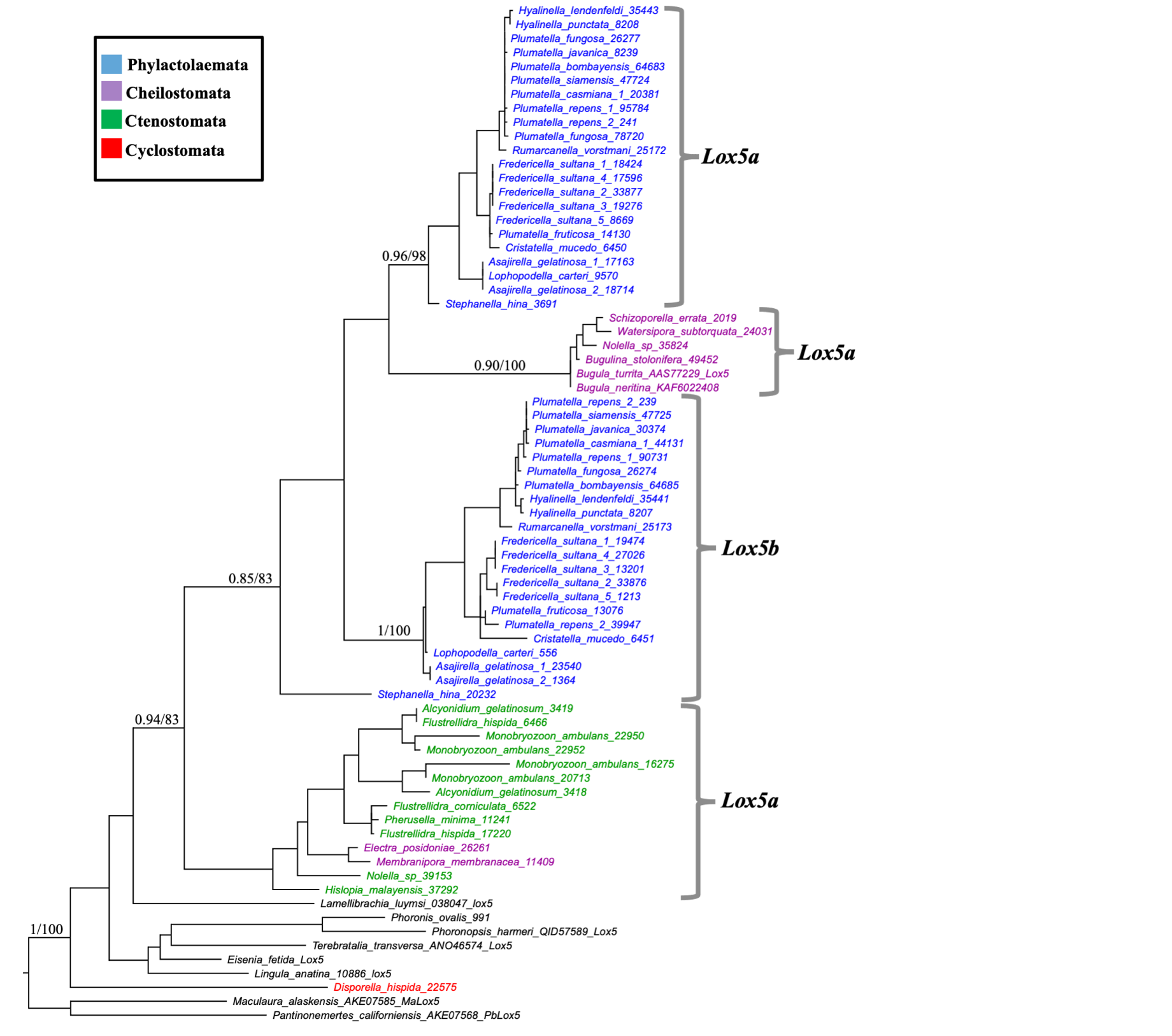


**Figure S5:** Expanded tree inference of *Lox5* gene (derived from Fig. 1). Bayesian posterior probabilities and bootstrap support values are shown for important clades, respectively. Accession numbers for NCBI database are displayed after the species names. Non bryozoan taxa were collapsed for an easier visualization.


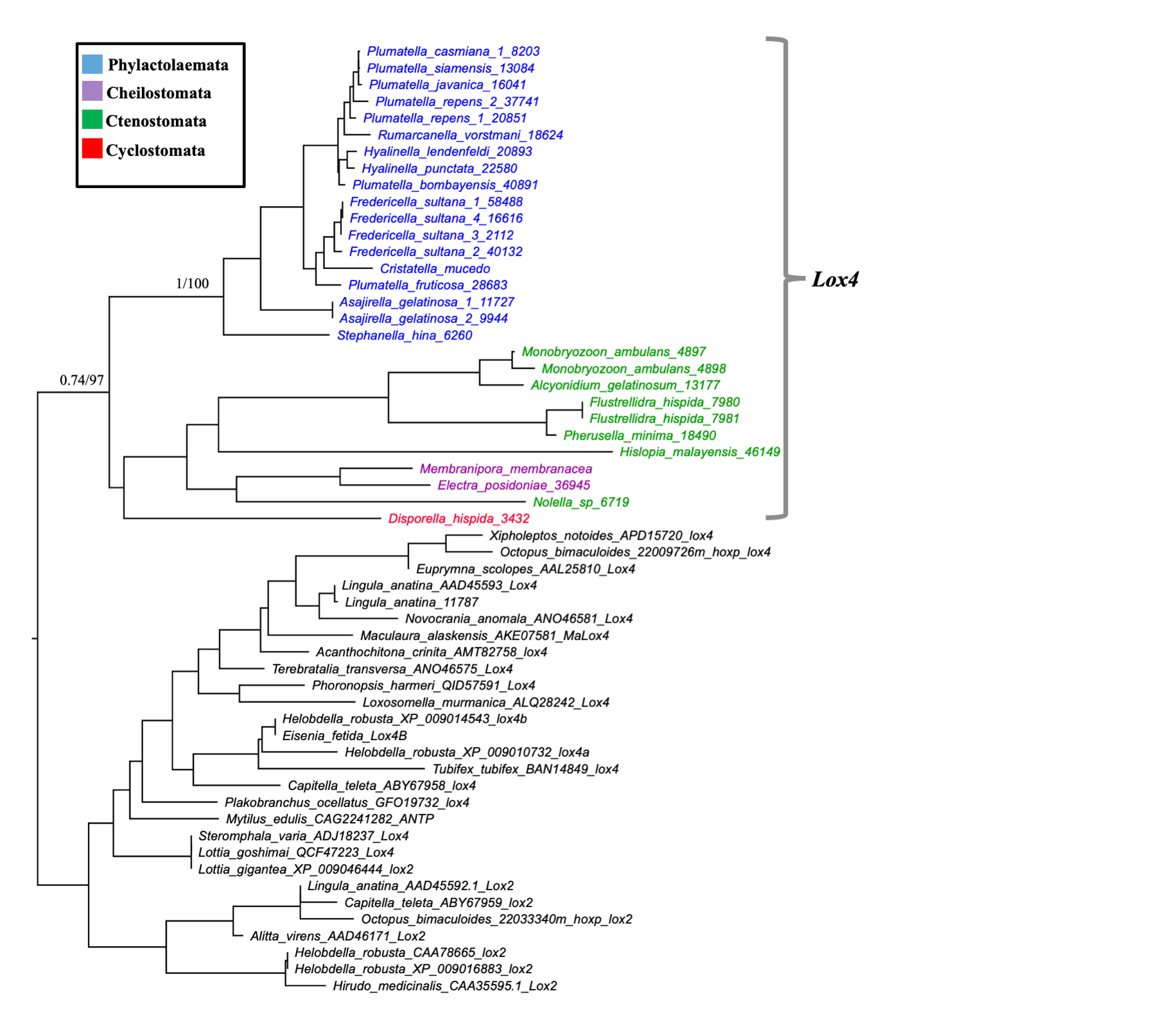


**Figure S6:** Expanded tree inference of *Lox4* gene (derived from Fig. 1). Bayesian posterior probabilities and bootstrap support values are shown for important clades, respectively. Accession numbers for NCBI database are displayed after the species names. Non bryozoan taxa were collapsed for an easier visualization.


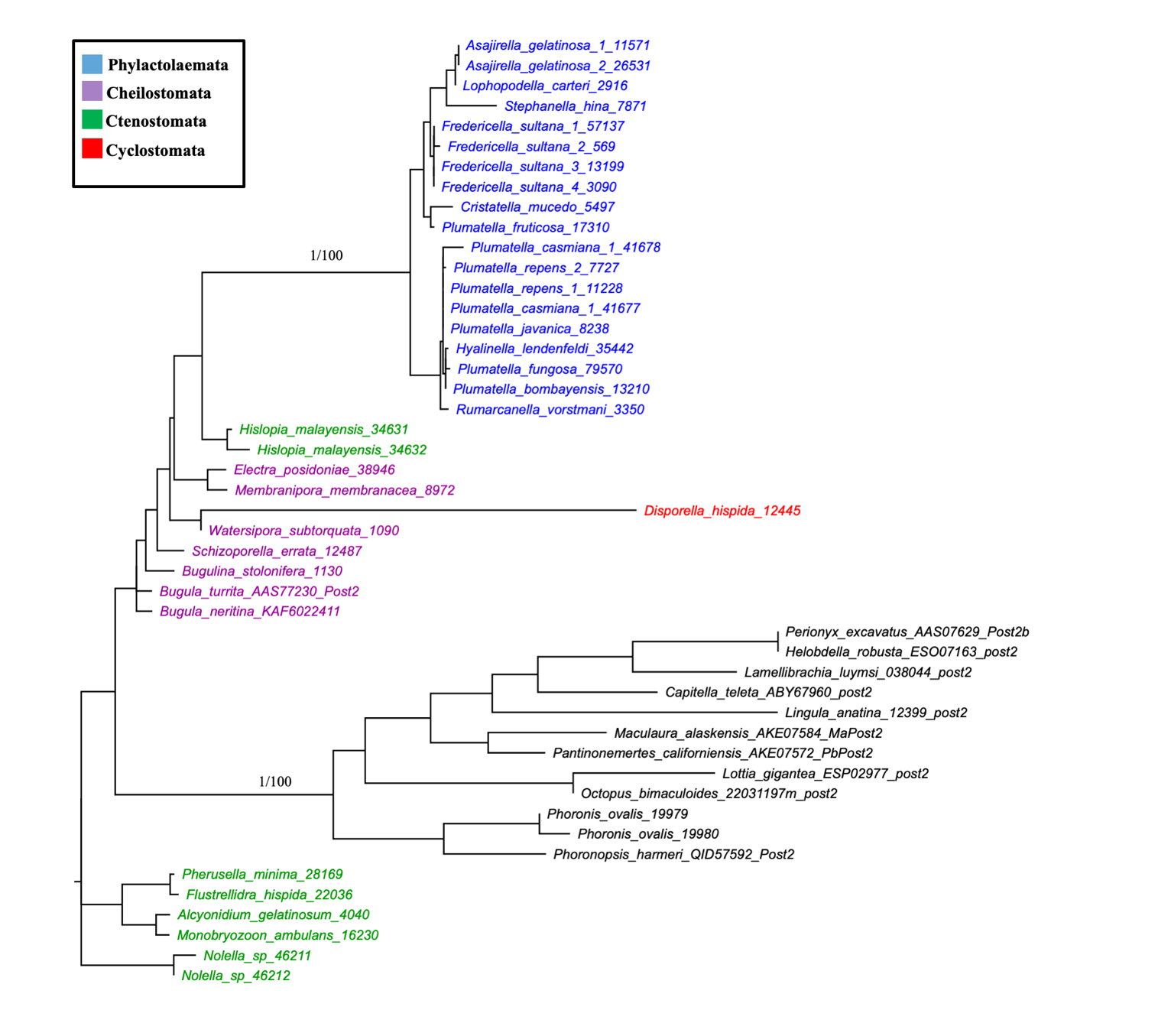


**Figure S7:** Expanded tree inference of *post2* gene (derived from Fig. 1). Bayesian posterior probabilities and bootstrap support values are shown for important clades, respectively. Accession numbers for NCBI database are displayed after the species names. Non bryozoan taxa were collapsed for an easier visualization.


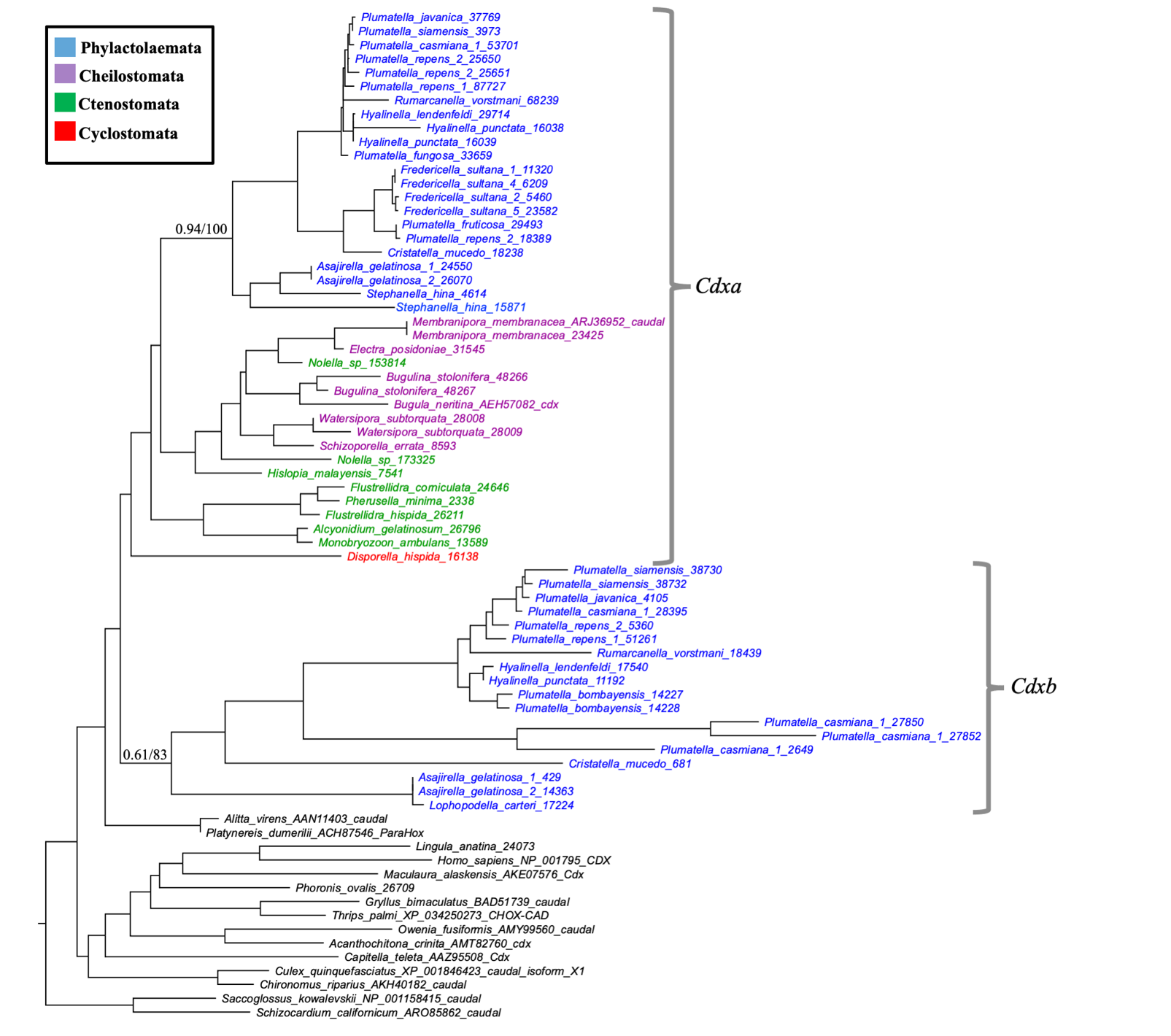


**Figure S8:** Expanded tree inference of *Cdx* gene (derived from Fig. 1). Bayesian posterior probabilities and bootstrap support values are shown for important clades, respectively. Accession numbers for NCBI database are displayed after the species names. Non bryozoan taxa were collapsed for an easier visualization.


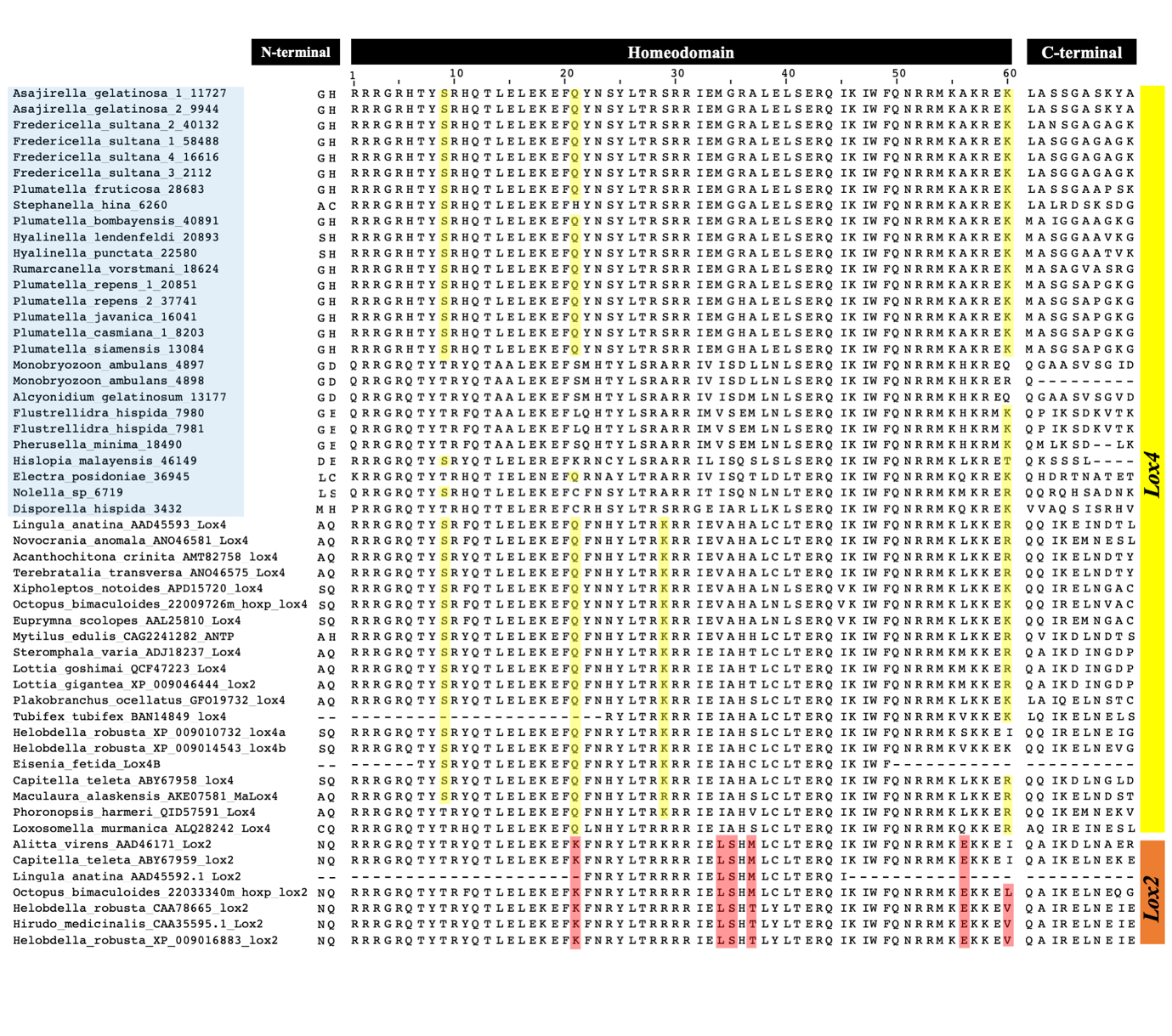


**Figure S9:** Multiple sequence alignment of *Lox4 and Lox2* genes showing the conserved homeodomain and flanking regions. Residues proposed as typical signatures of *Lox4* (de Rosa et al., 1999) are highlighted in yellow and typical signatures of *Lox2* are highlighted in light red. Bryozoans species names are present in the light blue box.


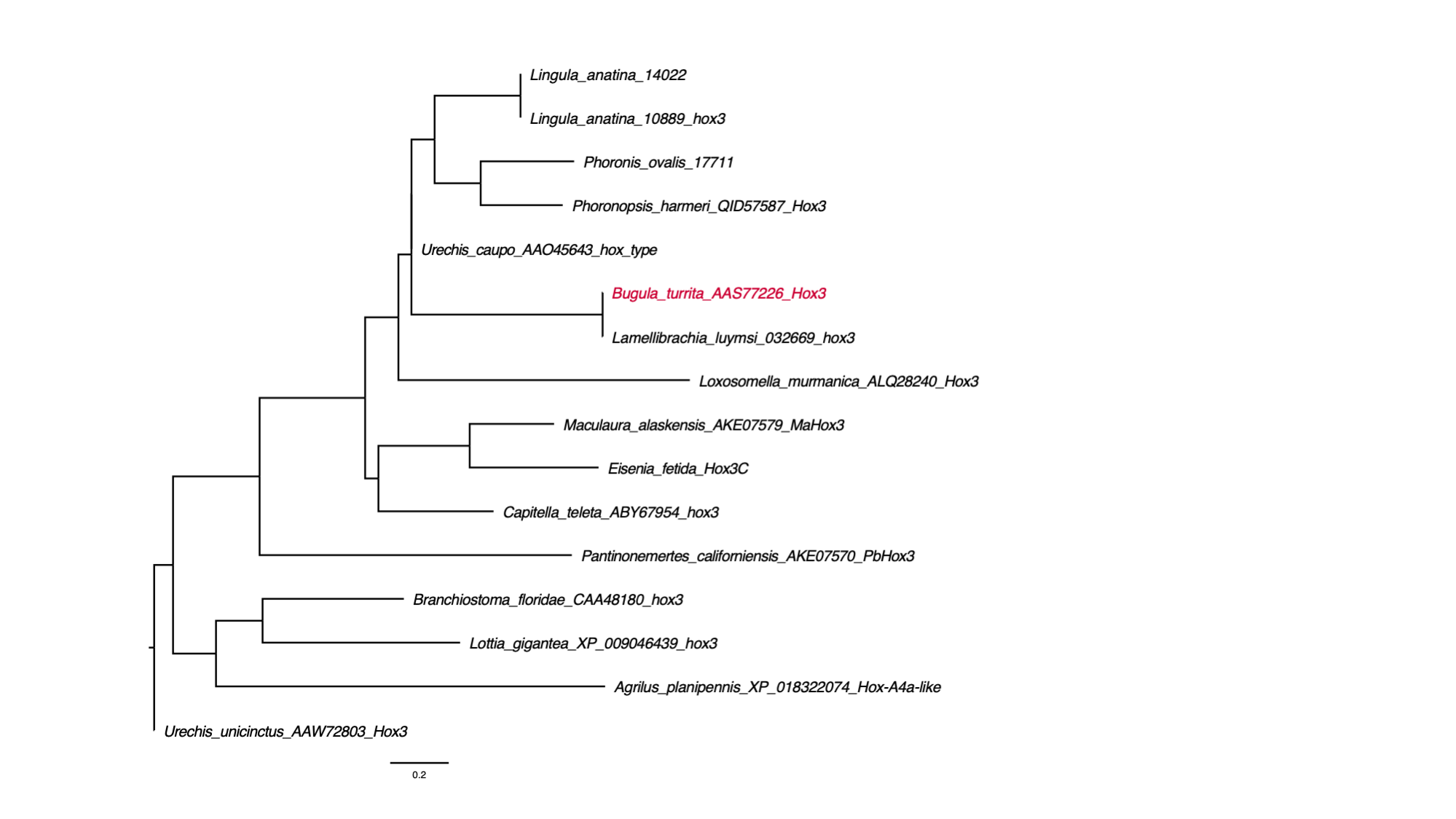


**Figure S10**: Expanded tree inference of *Hox3* gene (derived from Figure 1). Node support values represent Bayesian posterior probabilities and ultra-rapid bootstrap, respectively. Accession numbers for NCBI database are displayed after the spices names.

**References**

Bishop, J., Adkins, P., Wood, C., Jenkins, H., and Consortium, D.T.o.L. (2023). The genome sequence of the sea mat, Membranipora membranacea (Linnaeus, 1767). *Wellcome Open Research* 8(38)**,** 38.

Halanych, K.M., and Kocot, K.M. (2014). Repurposed transcriptomic data facilitate discovery of innate immunity toll-like receptor (TLR) Genes across Lophotrochozoa. *Biol Bull* 227(2)**,** 201-209. doi: 10.1086/BBLv227n2p201.

Kumar, G., Ertl, R., Bartholomew, J.L., and El-Matbouli, M. (2020). First transcriptome analysis of bryozoan Fredericella sultana, the primary host of myxozoan parasite Tetracapsuloides bryosalmonae. *PeerJ* 8**,** e9027-e9027. doi: 10.7717/peerj.9027.

Kutyumov, V.A., Predeus, A.V., Starunov, V.V., Maltseva, A.L., and Ostrovsky, A.N. (2021). Mitochondrial gene order of the freshwater bryozoan Cristatella mucedo retains ancestral lophotrochozoan features. *Mitochondrion* 59**,** 96-104. doi: 10.1016/j.mito.2021.02.003.

Laumer, C.E., Bekkouche, N., Kerbl, A., Goetz, F., Neves, R.C., Sørensen, M.V., et al. (2015). Spiralian phylogeny informs the evolution of microscopic lineages. *Curr Biol* 25(15)**,** 2000-2006. doi: 10.1016/j.cub.2015.06.068.

Laumer, C.E., Fernández, R., Lemer, S., Combosch, D., Kocot, K.M., Riesgo, A., et al. (2019). Revisiting metazoan phylogeny with genomic sampling of all phyla. *Proc Biol Sci* 286(1906)**,** 20190831. doi: 10.1098/rspb.2019.0831.

Luo, Y.J., Takeuchi, T., Koyanagi, R., Yamada, L., Kanda, M., Khalturina, M., et al. (2015). The Lingula genome provides insights into brachiopod evolution and the origin of phosphate biomineralization. *Nat Commun* 6**,** 8301. doi: 10.1038/ncomms9301.

Rayko, M., Komissarov, A., Kwan, J.C., Lim-Fong, G., Rhodes, A.C., Kliver, S., et al. (2020). Draft genome of Bugula neritina, a colonial animal packing powerful symbionts and potential medicines. *Scientific Data* 7(1)**,** 356. doi: 10.1038/s41597-020-00684-y.

Saadi, A.J., Bibermair, J., Kocot, K.M., Roberts, N.G., Hirose, M., Calcino, A., et al. (2022). Phylogenomics reveals deep relationships and diversification within phylactolaemate bryozoans. *Proc Biol Sci* 289(1986)**,** 20221504. doi: 10.1098/rspb.2022.1504.

Santagata, S. (2021). Genes with evidence of positive selection as potentially related to coloniality and the evolution of morphological features among the lophophorates and entoprocts. *J Exp Zool B Mol Dev Evol* 336(3)**,** 267-280. doi: 10.1002/jez.b.22975.

Treibergs, K.A., and Giribet, G. (2020). Differential Gene Expression Between Polymorphic Zooids of the Marine Bryozoan Bugulina stolonifera. *G3 (Bethesda)* 10(10)**,** 3843-3857. doi: 10.1534/g3.120.401348.

Wood, C., Bishop, J., Adkins, P., Jenkins, H., and Consortium, D.T.o.L. (2023). The genome sequence of an erect bryozoan, Bugulina stolonifera (Ryland, 1960). *Wellcome Open Research* 8(26)**,** 26.
